# Use of Hospital Information System to Improve the Quality of Health Care from Clinical Staff Perspective

**DOI:** 10.31661/gmj.v10i0.1830

**Published:** 2021-11-28

**Authors:** Ali Mohammadpour, Mohammad Mehdi Ghaemi, Reza Darrudi, Hassan Ebrahimpour Sadagheyani

**Affiliations:** ^1^School of Para Medicine, Hamadan University of Medical Sciences, Hamadan, Iran; ^2^School of Management and Information Science, Kerman University of Medical Sciences, Kerman, Iran; ^3^Department of Health Information Technology, Neyshabur University of Medical Sciences, Neyshabur, Iran

**Keywords:** Hospital Information System, Performance Improvement, Quality of Health Care, Clinical Staff’s Perspective

## Abstract

**Background:**

The development of hospital information systems (HISs) has a significant effect on care processes. In this regard, the clinical staff’s perspective is very important in evaluating the success of these systems. The present study aimed to evaluate the clinical staff’s perspective at hospitals affiliated to Neyshabur University of Medical Sciences on the effectiveness of the HIS in improving their performance and strategies for increasing the system effectiveness.

**Materials and Methods:**

In the present cross-sectional study, 120 clinical staff who actively worked with the HIS were included. A two-part questionnaire was used for data gathering.

**Results:**

From the perspective of the research population, the effective score of HIS was %64.42 in improving their performance, and %81.85 in the case of developed HIS according to the suggested strategies. According to the research scale, the effectiveness of the system could be improved from good to excellent in the case of implementation of strategies, so that there was a statistically significant difference between the pre- and post-effectiveness (P<0.001).

**Conclusion:**

Positive population perspective on the effectiveness of HIS in-performance improvement of the clinical staff indicated that there was a good context for the development and utilization of information technology in the hospitals. The clinical staff’s opinions and work needs as the primary group of patient care should be taken into consideration in order to increase the effectiveness of the HIS.

## Introduction

Information technology (IT) has changed various aspects of human life and brought about fundamental changes in it [1,2,3]. The health sector is an area that is affected by IT[1,3,4,5]. The process of collecting and accessing health information is the most influential dimension in health [6]. Since providing health care for society is very complex and closely linked to information, it is impossible to ignore information and communication technology in medical and health care, especially hospital information systems (HIS) [7]. The HIS is a system that provides the process of collecting, storing, processing, retrieving, and displaying information needed for hospital education, management, and research [8]. The main purpose of this system is to support hospital activities at the practical, tactical, and strategic levels to provide better service for patients [9]. In general, some studies have indicated the impact of using these systems in the health care system [10,11]. For instance, studies have indicated that health service-providing systems utilize significant benefits such as reduction of patient waiting time [12], reduction of mortality [13,14], drug side effects management [15,16], health care professionals’ prompt and timely access to up-to-date patient information [12,17,18], reduction of medical errors [12,19], optimal service management [20,21], and improvement of care processes up to 60% [22]. Although some studies have positively evaluated the impact of the HIS, it is important to note that achieving the aforementioned benefits requires considering different infrastructures, including major users’ views in the system. In other words, the system needs to be accepted by the main users [11] because the system rejection ultimately leads to the effectiveness and performance of the HIS. The performance of any information system refers to a degree of organizational effectiveness that is achieved through the information system; hence, improving the HIS performance implies that the information system performance is tailored to internal and external changes and users’ different demands [3]. Cilliers and Flowerday (2013) found that 72% of HIS users believed that telemedicine helped improve the quality of their work. In this regard, users in rural areas used the information system and were more satisfied than users who worked in urban care centers, reflecting the appropriateness of the system’s performance to work environment processes and users’ demands in the information system [23]. All hospitals in Iran have HISs, but these software are manufactured by different companies [24]. The HIS of Neyshabur University of Medical Sciences (Version 8) was obtained from Tirageh-Computer Co. in Tehran in 2009 and was first implemented at Hakim Hospital and is currently being used in both Hakim and 22-e-Bahman hospitals. HISs of Neyshabur University of Medical Sciences have subsystems including electronic patient record management, hospital financial management procedures, evidence-based decision support systems, patient scheduling, paraclinical subsystems, and ward management [6,23]. Given that the hope for the system's effetiveness in improving care for hospital clinical staff indicated the belief that the use of the system helped them achieve their career goals and made them successful in providing quality care. There was no study on HIS in hospitals affiliated to Neyshabur University of Medical Sciences. Hence, the present study aimed to investigate the impact of the HIS on the improvement of clinical staff performance and strategies to increase its effectiveness from the perspective of clinical staff.

## Materials and Methods

**Figure 1:**
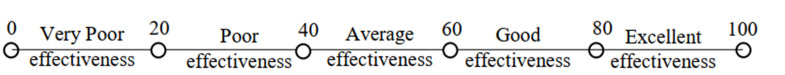
Levels of measurement and scaling

The present cross-sectional study was conducted in Neyshabur in 2019. The research population consisted of 120 clinical staff at Hakim and 22-e-Bahman hospitals of Neyshabur University of Medical Sciences (physicians, nurses, staff of laboratory sciences, midwifery, radiology, anesthesia, operating room, and health IT) who used the HIS. The census method was used in the present study; hence, all clinical and care staff who were bachelor and higher than bachelor degree used the HIS at least two days a week and were directly involved in patient care were included. Three nurses and two physicians were excluded due to lack of willingness to continue cooperation in the study. Neyshabur University of Medical Sciences has two teaching-clinical hospitals. It has used the manufactured HIS by Tirageh Computer Co. of Tehran since 2009. The data collection tool was a researcher-made questionnaire based on valid scientific literature [2,13,][25], medical informatics expert’s opinion, health IT, and available indices in a model by Ahitof and Newman [26]. The present questionnaire consisted of two parts; first, about demographic information of the research population (age, sex, education degree, field of study, work experience, and status of users’ training courses). Second, with 43 two-part questions; first, for examining the research population’s view about the impact of the HIS on improving the clinical staff performance (therapeutic, educational, research, and management processes in the hospital domain); and the second part about the importance of that item as an effective feature in enhancing the effectiveness of the HIS in the further improvement of performance. The reliability of the questionnaire was measured by the Cronbach’s alpha coefficient (α=97%) and its validity by the content validity. The questionnaire items included the 5-point scale (Very Low, Low, Medium, High, and Very high) with scores of 1 to 5, respectively (Figure-1). The effective score of HIS as the evaluation criterion on the improvement in clinical staff performance was calculated by the following formula: Effectiveness Index = {Σ (WtA × A + WtB × B +…WtN * N)}/ (N × 5) Where: A, B… N are the mean effectiveness ratings to survey questions on the main factors. WtA…N are relative importance weights given by clinicians to each of the main factors. N is the number of main factors. Number 5 is the highest score of any item. The following scale was used for to further interpretation of effective score of each factor on the performance improvement and also as a solution to improve the effectiveness of the HIS. The normality of the explanatory variables was checked by using the Shapiro-Wilk test. This study was approved by the Research Ethics Committee Neyshabur University of Medical Sciences under Opinion number IR.NUMS.REC.1398.004. Questionnaires were completed in person at the workplace of the research population. The results were reported according to descriptive statistics indices including mean and standard deviation; and the Paired T-Test, ANOVA, and the Pearson correlation coefficient by SPSS Version 25 (SPSS Inc. Chicago, Illinois, USA) were used to compare groups at a significant level of 0.05.

## Results

**Figure 2:**
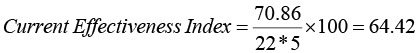
The formula of Current Effectiveness Index calculation

**Figure 3:**
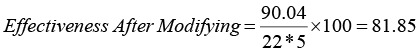
Formula for calculating the effectiveness index after applying changes

 Based on research findings, most (80%)HIS users were women. The mean age of participants was 33.85±8.2 years, and most of them were in the age group of 20-39, and 33 (27.5%) had less than five years of work experience. Ninety-five (79.17%) had bachelor's degrees, and 48 (40%) were nurses. Among them, 15 (12%) physicians used the system less than the rest. Also, 45 (37.5%) were users of computer training courses or HIS (Table-1). According to the research findings, there was a statistically significant difference between mean current effectiveness score (3.22) and the score of HIS effectiveness after developing factors as strategies for increasing the effectiveness of the HIS (4.09). In addition, with the development of the factors considered as strategies to increase the efficiency of HIS, the performance of clinical staff will improve (P<0.001, r=0.7). Also, based on the results of the current and developed effect difference column of the HIS, the HIS was far from ideal in terms of information integration (diff [BA]=1.18) and establishing telemedicine (diff [BA]=1.12, Table-2). According to the research population view, the highest effect of the HIS on improving their performance (improving therapeutic, educational, research, and management processes) belonged to accelerating paraclinical processes (mean=3.62), access to information (mean=3.43), and medical research (mean=3.65). The effectiveness score of the HIS on staff performance improvement was calculated to be 64.42% (Figure-2) that was good based on the research scale (Table-2). From the perspective of the research population, formulating specific rules for the acceptance of computer documentation in judicial authorities (mean=4.53), cooperation between IT experts, other staff, especially physicians and nurses (mean=4.33), facilitating education and research, simplifying paraclinical processes (mean=4.28), and the system, and defining user needs and priorities (mean=4.23), and computer literacy, and holding related HIS courses (mean=4.16) were the important factors in increasing the effectiveness of the HIS in improving their performance. The effectiveness score of the HIS on the staff performance was calculated to be 81.85% (Figure-3) by developing factors. The HIS effectiveness was high based on the research scale (Table-2). According to the relationship between the research population’s view on the effectiveness of the HIS in improving staff performance, there were significant relationships between research population jobs and medical research facilitation factors (P<0.001), formulating specific rules on the acceptance of computerized documentation in judicial authorities (P=0.001), integrating hospital systems (P=0.002), patient safety (P=0.004), identifying and defining user preferences and needs (P<0.001), identifying problems, obstacles and application of IT (P=0.028), and appropriate foresight (P=0.001), private sector partnerships, maintaining the data confidentiality and security(P<0.001), the collaboration of IT experts with other staff, especially physicians and nurses (P<0.001), and the ability to learn and training continues to improve care (P=0.001). In the examination of the relationship between the research population’s view on the effectiveness of HIS in improving the staff performance, a significant relationship was only found between gender and telecommunications facilities (P<0.001). In terms of each factor as a way to increase effectiveness, everyone had the same views except for cooperation of the private sector (P=0.001), and the collaboration between IT experts and other staff, especially physicians and nurses (P<0.001); there was no significant difference (Table-3). According to the investigation the relationship between the research population’s view on the effectiveness of the HIS in improving the clinical staff performance and the health care system and having computer-related training courses and the HIS, there was a significant relationship only in influencing the medical research (P<0.001), enhancing patient safety (P<0.003) and identifying problems, barriers and application of IT, and proper foresight (P<0.042). The clinical staff had no similar view on the extent of effectiveness of specific laws in the adoption of computerized documentation in judicial authorities (P=0.047) and the private sector participation in the development of the HIS to increase the effectiveness of HIS (P<0.001), integration of health information (P=0.031), and the collaboration between IT experts and other staff, especially physicians and nurses (P<0.001). There was no significant difference in terms of the effectiveness of other factors (Table-3). Regarding Table-4, physicians had a more positive view on the current effect of HIS on the improvement of performance and health care system than other staff; and they reported the importance of factors. Paramedics and nurses evaluated the impact of the HIS on the performance improvement and quality of care. Furthermore, nurses had a more emphasis on improving their performance by improving the examined factors. Also, Table-4 indicated a significant difference between the research population's views on the effectiveness of the HIS in improving the staff performance and the health care system (therapeutic, educational, research, and managerial processes) and the importance of the effectiveness of factors in enhancing the clinical staff performance and the health care system through the HIS (P<0.001); and the correlation coefficient between the effect of factors and their importance was r=0.78 in the nursing group, r=0.69 in paramedics and IT staff, and r=0.26 in physicians (Table-4).

## Discussion

Despite great effort to develop and utilize HISs and improve their effectiveness in improving patient care, the present study indicated an index of 64.42 out of 100 and reflected that there was still a gap to complete effectiveness. In a study by Itumalla (2012) in Indian hospitals, the customer satisfaction with the effectiveness of the HIS in improving the quality of care was 75.87 [2], reflecting the fact that we were weak in the use of information systems. However, the effectiveness could reach the index of 81.85 out of 100 by utilizing the solutions provided by HISs users. Many studies have emphasized the positive impact of HISs on cardiac care, intensive care, medication administration, and nursing care [27,28,29]. The present study examined and analyzed the factors and indicated the most remarkable effectiveness of the HIS in improving the clinical staff performance occurred by accelerating paraclinical activities, the process of diagnosis and treatment, access to quality information, and medical research. The results of some other studies also confirmed the findings of the present study [11,30,31]. According to findings of the present study, the possibility of using the telemedicine and providing facilities in the HIS to increase the patient safety such as the possibility of using simulators to train students and the ability to measure patients’ vital symptoms through wireless sensors were important factors that played insignificant roles in improving patient care, indicating the fact that the HIS surveys needed upgrading telemedicine and providing facilities for patient safety. In this regard, Cilliers and Flowerday stated that only 34% of health centers in South Africa used telemedicine. They stated that the staff’s lack of knowledge and awareness of telemedicine was the most important obstacle to the implementation and effective implementation use of telemedicine [23]. What is important is the influence of research factors on increasing the effectiveness of HIS and improving the clinical staff performance. Based on our findings, two factors, namely the formulation of specific laws for accepting computerized documentation in judicial authorities and the IT expert collaboration with other staff, especially physicians and nurses, to optimize the HIS, could significantly impact the clinical staff performance. In the field of computerized document acceptance, the acceptance of these documents by judicial authorities could prevent many processes from being reworked and thus save time for staff to do care. Therefore, the Ministry of Health needs to adopt laws and mechanisms to resolve the problem. According to our results, physicians had more positive views on the effectiveness of the HIS in improving their performance than other staff and reported the greater effectiveness of the HIS in medical research. Sadoughi et al.(2017) found that according to physicians, the greatest effectiveness of the HIS in improving care was due to its use in medical research, and it was consistent with findings of the present study [32]. Karami also stated that smart systems could be considered as a tool for accessing the latest medical findings to make the best decisions for treating patients. Furthermore, the access to all patient information avoided many reworks and accelerated the process of treatment and diagnosis [28]. The willingness to consult and receive self-care training in disease management [33][34] is the motivation for telemedicine and teleconsultation, which are major applications of health IT [35] and play important roles in accelerating service delivery, reducing costs [36], and subsequently increasing productivity. On the other hand, concepts of expediting service delivery, reducing costs, and increasing productivity are somehow related. Increased productivity can be due to lower costs due to the use of IT [37]. The use of telemedicine and teleconsultation can play important roles in expediting service delivery that inevitably result in lower service costs [23]. In the present study, the findings also confirmed the effectiveness of the HIS on the above cases. According to findings of the present study, According to findings of the present study, from the perspective of the research population, the integration of HIS, as an important factor in increasing the effectiveness of the HIS to improve the clinical staff’s performance, was far from ideal; hence, the IT professionals should pay more attention to this issue as the information sharing, and electronification of health services needed a secure-integrated environment [38]. In examining the relationship between the research population’s view on the effectiveness of the HIS on the staff performance improvement, it was found that nurses significantly considered a higher gap between the current and optimal status; and it was consistent with results of a study by Jebraeily et al. [39]. Since nurses are the main clinical users of the HIS, they play important roles in documenting and performing patient care; hence, adopting IT and meeting their business needs in HIS processes can have a significant impact on the development of these technologies and pave the way for better utilization of these systems.The familiarity and use of a small number of physicians as care managers with the HIS and participation of HIS users involved in patient care were the most important weak and power points of the present study, respectively.

## Conclusion

 The positive viewpoint of the research population on the effectiveness of the HIS in improving the clinical staff’s performance and the belief in improvement of the system by developing the proposed factors indicated that there was a good context for the development and exploitation of ITs in the hospitals, and projects such as hopeful health electronic records could be planned for its development and utilization. The effectiveness of HIS can increases in improving the performance of clinical staff and the health care system by creating an appropriate organizational culture and providing adequate training for therapists as key users of these systems, and paying attention to their work needs in the HIS. Since the implementation, implementation, and support of such systems are very costly, it is important to pay attention to the importance of budgeting in this field and take necessary measures to ensure the full acceptance of the documents derived from these systems in the legal assemblies.

## Acknowledgment

 Hereby, the authors sincerely thank and appreciate all of those who collaborated with the authors during this research and all the authorities of Neyshabur University of Medical Sciences, Iran. This study was funded by the Neyshabur University of Medical Sciences, Iran (grant number: 97282).

## Conflict of Interest

 The authors declare that there is no conflict of interest.

**Table 1:** Frequency Distribution of Demographic Characteristics of the Research Population.

** Demographic characteristics**D	** N(%)**
**Sex **	
Female	96(80)
Male	24(20)
** Age Group (year)**	
20-29	48(40)
30-39	48(40)
40-49	21(17.5)
>50	3(2.5)
** Education level**	
Associate Degree	10(8.33)
Bachelor	95(79.17)
Ph.D.	15(12.5)
**Occupation status **	
Physicion	15(12.5)
Nurse	48(40)
Paramedical and health IT	57(47.5)
** Work experience(year)**
<5	33(27.5)
5-15	57(47.5)
15-25	24(20)
>25	6(5)
**Attend computer and IT workshops **
Yes	75(62.5)
No	45(37.5)

**Table 2:** Research Population Attitude Towards the Effect of HIS on Improve Their Performance and Increasing the Effectiveness of HIS if the Studied Factors are Developed.

** NO **	** Factors **		**Current Effectiveness and Importance Ratings**						
		**Mean Current Effectiveness Score [A]**	** Mean Importance Ratings Score [B] **	** Relative Importance Weight C=(B/ΣB) **	** Relative Effectiveness weight D=(A/ΣA) **	** Relative Score (Effectiveness) E=A×C×N **	** Relative Score (Importance) F=B×C×N **	** Deference [B-A] **
1	** Access to patient information **	3.43	4.12	0.05	0.05	3.65	4.39	0.69
2	** Accelerating diagnosis and treatment **	3.19	3.94	0.05	0.05	3.16	4.02	0.75
3	** Incidence of medical errors **	3.35	4.16	0.05	0.05	3.48	4.48	0.81
4	** Telemedicine **	2.88	4	0.04	0.05	2.58	4.14	1.12
5	** Unnecessary patient admissions and facilitating patient admission and discharge **	3.26	4.02	0.05	0.05	3.3	4.18	0.76
6	** Facilitating paraclinical processes (lab test, radiology, support consultations) **	3.62	4.28	0.05	0.05	4.07	4.74	0.66
7	** Facilitate nursing care processes **	3.26	4.01	0.05	0.05	3.30	4.16	0.75
8	** Medical research **	3.65	4.28	0.05	0.05	4.14	4.74	0.63
9	** Develop specific rules for the acceptance of HIS documentation in the judicial authorities **	3.51	4.53	0.05	0.05	3.83	5.31	1.02
10	** Reducing costs and Increasing productivity of equipment and facilities **	3.2	3.89	0.05	0.05	3.18	3.92	0.69
11	** Integration of health information **	2.96	4.14	0.04	0.05	2.72	4.44	1.18
12	** Production of information resources **	3	3.87	0.04	0.05	2.79	3.88	0.87
13	** Patient Safety **	2.87	3.78	0.04	0.04	2.56	3.7	0.91
14	** Computer literacy and HIS **	3.25	4.18	0.05	0.05	3.28	4.52	0.93
15	** Increase in clinician motivation **	3.15	4.13	0.04	0.05	3.08	4.42	0.98
16	** Providing clinician and End users work needs**	3.33	4.23	0.05	0.05	3.44	4.63	0.9
17	** Identifying and troubleshooting software HIS errors.**	3.2	4.13	0.05	0.05	3.18	4.42	0.93
18	** Private enterprise participation in HIS project development**	3	3.93	0.04	0.05	2.79	4	0.93
19	** Updating HIS**	3.33	4.23	0.05	0.05	3.44	4.63	0.9
20	** Alteration and overwriting data and violation of legal rights of patients**	3.2	3.93	0.05	0.05	3.18	4	0.73
21	Collaboration between health IT* experts and physicians and nurses to advance HIS goals	3.2	4.33	0.05	0.05	3.18	4.85	1.13
22	** Distance and continuing education**	3.02	3.93	0.04	0.05	2.83	4	0.91
	** SUM**	70.86	90.04	1	1	71.16	95.58	19.18
	** Mean**	3.22	4.09	-	-	3.23	4.34	0.64
	** P-Value**	0.000	0.000
	** r**	0.7	0.7

**Table 3:** Research Population’s Attitude towards the Effectiveness of HIS in Improving Clinical Staff Performance and Increasing the Effectiveness of HIS Based on the Examined Factors in Terms of Demographic Characteristics.

** NO**	** Factors**		** Demographic Features **	
		** Current Effectiveness (P-Value)**			** Importance Ratings (P-Value)**		
		** Sex**	** Occupation**	** Workshop**	** Sex**	** Occupation**	**Workshop**
1	**Access to patient information**	0.316	0.239	0.437	0.484	0.059	0.603
2	**Accelerating diagnosis and treatment**	0.408	0.142	0.394	0.527	0.032	0.158
3	** Incidence of medical errors**	0.746	0.205	0.625	0.1	0.14	0.365
4	** Telemedicine**	0.000	0.109	0.554	0.265	0.000	0.294
5	**Unnecessary patient admissions and facilitating patient admission and discharge**	0.309	0.071	0.394	0.18	0.008	0.168
6	**Facilitating paraclinical processes (lab test, radiology, support consultations)**	0.499	0.392	0.329	0.591	0.293	0.468
7	**Facilitate nursing care processes**	0.885	0.437	0.29	0.361	0.027	0.334
8	** Medical research**	0.445	0.000	0.000	0.23	0.000	0.104
9	** Develop specific rules for the acceptance of HIS documentation in the judicial authorities**	0.453	0.001	0.869	0.777	0.005	0.047
10	**Reducing costs and increasing productivity of equipment and facilities**	0.28	0.184	0.613	0.306	0.04	0.252
11	**Integration of health information**	0.155	0.002	0.207	0.329	0.02	0.031
12	**Production of information resources**	0.61	0.208	0.159	0.7	0.096	0.486
13	**Patient safety**	0.387	0.004	0.003	0.072	0.022	0.102
14	** Computer literacy and HIS**	0.151	0.934	0.188	0.119	0.052	0.482
15	** Increase in clinician motivation**	0.535	0.316	0.309	0.462	0.000	0.738
16	** Providing clinician and end users work needs**	0.661	0.000	0.653	0.36	0.002	0.401
17	** Identifying and troubleshooting software HIS errors.**	0.828	0.028	0.042	1	0.093	0.264
18	** Private enterprise participation in HIS project development**	0.775	0.001	0.073	0.001	0.000	0.000
19	** Updating HIS**	0.507	0.088	0.387	0.188	0.002	0.931
20	** Alteration and overwriting data and violation of legal rights of patients**	0.655	0.000	0.378	0.122	0.000	0.086
21	** Collaboration between health IT experts and physicians and nurses to advance HIS goals**	0.828	0.000	0.661	0.000	0.011	0.000
22	** Distance and continuing education**	0.176	0.001	0.215	0.313	0.000	0.494

**Table 4:** Statistical Correlation Indices in Research Groups in Terms of Job.

** Jobs**	** Current effectiveness (Mean Value)**	** Importance ratings (Mean Value) **	** Mean difference**
** Paramedical and health IT**
Mean±SD	3.25±0.45	3.88 ±0.33	0.63
P-Value		0.001	
r		0.69	
** Nurse**		
Mean±SD	3.02 ±0.34	4.16 ±0.3	1.14
P-Value		0.002	
r		0.78	
** Physician**		
Mean±SD	3.40±0.36	4.34±0.25	0.94
P-Value		0.000	
r		0.26
